# The contribution of ion channels to shaping macrophage behaviour

**DOI:** 10.3389/fphar.2022.970234

**Published:** 2022-09-07

**Authors:** Anna Selezneva, Alasdair J. Gibb, Dean Willis

**Affiliations:** Department of Neuroscience, Physiology, and Pharmacology, University College London, London, United Kingdom

**Keywords:** macrophage, ion channels, immune signalling, innate immunity, membrane potential (ΔΨ), inflammation

## Abstract

The expanding roles of macrophages in physiological and pathophysiological mechanisms now include normal tissue homeostasis, tissue repair and regeneration, including neuronal tissue; initiation, progression, and resolution of the inflammatory response and a diverse array of anti-microbial activities. Two hallmarks of macrophage activity which appear to be fundamental to their diverse cellular functionalities are cellular plasticity and phenotypic heterogeneity. Macrophage plasticity allows these cells to take on a broad spectrum of differing cellular phenotypes in response to local and possibly previous encountered environmental signals. Cellular plasticity also contributes to tissue- and stimulus-dependent macrophage heterogeneity, which manifests itself as different macrophage phenotypes being found at different tissue locations and/or after different cell stimuli. Together, plasticity and heterogeneity align macrophage phenotypes to their required local cellular functions and prevent inappropriate activation of the cell, which could lead to pathology. To execute the appropriate function, which must be regulated at the qualitative, quantitative, spatial and temporal levels, macrophages constantly monitor intracellular and extracellular parameters to initiate and control the appropriate cell signaling cascades. The sensors and signaling mechanisms which control macrophages are the focus of a considerable amount of research. Ion channels regulate the flow of ions between cellular membranes and are critical to cell signaling mechanisms in a variety of cellular functions. It is therefore surprising that the role of ion channels in the macrophage biology has been relatively overlooked. In this review we provide a summary of ion channel research in macrophages. We begin by giving a narrative-based explanation of the membrane potential and its importance in cell biology. We then report on research implicating different ion channel families in macrophage functions. Finally, we highlight some areas of ion channel research in macrophages which need to be addressed, future possible developments in this field and therapeutic potential.

## Introduction

Belonging to the innate branch of the immune system, macrophages are important in the killing of invading microbes, the removal of noxious material; the initiation, propagation and resolution of inflammatory responses, the progression of tissue repair and the maintenance of homeostasis (Murray and Wynn, 2011; Wynn et al., 2013). However, an inappropriate, exaggerated or dysfunctional macrophage response can lead to host tissue damage and contribute to a variety of pathologies (Belchamber and Donnelly, 2017; Crayne et al., 2019). To carry out their required biological functions macrophages need to produce qualitatively, quantitatively and temporally the appropriate biomolecules. They achieve this by utilizing a complex series of interconnected, feedback rich, signalling cascades (Raza et al., 2008; Drexler et al., 2008). These signalling pathways receive input from numerous cellular sensors, which allow the macrophage to respond in a timely and appropriate manner to extracellular and intracellular signals, which are often in a state of constant flux, to produce the appropriate response. Indeed, Mosser *et al.* has recently referred to macrophages as “transducers of the body” (Mosser et al., 2020). This diversity of functions and flexibility of response to environmental cues results in macrophages showing phenotypic heterogeneity. How macrophage populations are classified is highly debatable and depends on various criteria including tissue location/lineage, current cellular functionality or activation state and the granularity of the classification profiles used. However, a convenient, if simplified, way to classify them is pro-inflammatory M1 macrophages and anti-inflammatory M2 macrophages (Smith et al., 2016; Gordon and Plüddemann, 2017; Gessain et al., 2020).

Ion channels control the movement of ions, such as Na^+^, K^+^, and Ca^2+^, across cell membranes, both plasma and intracellular membranes such as mitochondrial, lysosomal, endoplasmic reticulum membranes and nuclear membranes, and are known to be important cellular sensors and participate in cell signalling cascades in numerous cell types (Selezneva et al., 2022).

Here we will provide an up-to-date overview of ion channels known to be present and functional in macrophages. First, we give a brief introduction to membrane potential and how it could impact macrophage biology. Then, beginning with potassium (K^+^) channels, which are the main regulators of cell membrane potential, we review the evidence which supports a role for ion channels, Table 1, in shaping macrophage behaviour. Finally, we discuss prospects in this area of research and the potential of macrophage ion channels as therapeutic targets.

**TABLE 1:** Currently identified functional ion channels in macrophages.

Channel	Permeability	Subcellular localization	Modulation of activity	Functions in macrophages
Potassium (See Table 2 for individual channel types)	K^+^	Plasma membrane, nuclear envelope, endolysosomes	Voltage-gated and/or Ca^2+^ activated, lipid-gated, pH, membrane stretch	Polarization, migration (Chung et al., 2002) (Toyama et al., 2008), transcriptional activity, cytokine production (Hanley et al., 2004) (Yoshida, 2017) (Selezneva et al., 2021), phagocytosis (Sun et al., 2020), inflammasome activation (Wu et al., 2021) (Di et al., 2018)
Transmembrane Protein 175 (TMEM175)	K^+^	Endolysosomes	Conformational changes in Akt kinase	Autophagosome-lysosome fusion (Cang et al., 2015)
Sodium (Na_v_1.1, Na_v_1.3–1.7 and Na_v_1.9)	Na^+^	Late endosomes, phagolysosomes, cytoskeletal filaments, endoplasmic reticulum	Voltage-gated	Phagocytosis (Carrithers et al., 2011) (Craner et al., 2005), endosomal acidification (Carrithers et al., 2007), inflammatory mediator release (Montero et al., 2020)
Acid-Sensing Ion Channel (ASIC) (ASIC1, ASIC2a, ASIC3)	Na^+^	Nuclei, cytoplasm	Extracellular H^+^	Migration, endocytosis, cytokine production, apoptosis (Ni et al., 2018) (Kong et al., 2013)
Calcium Release-Activated Channel (CRAC)	Ca^2+^	Plasma membrane	Depletion of endoplasmic reticulum Ca^2+^ store	Ca^2+^ signalling (Gao et al., 2010), phagocytosis (Braun et al., 2009), cytokine production (del Corno et al., 2001)
L-type Calcium	Ca^2+^	Plasma membrane	Voltage-gated	Ca^2+^ signalling, responses to infection (Antony et al., 2015) (Sharma et al., 2016)
Inositol 1,4,5-trisphosphate receptor (IP3R)	Ca^2+^	Endoplasmic reticulum	IP_3_	Ca^2+^ signalling (Sampieri et al., 2018), polarization (Liu et al., 2019), cytokine production (Staats et al., 2016)
Two-Pore Channel (TPC)	Na^+^, Ca^2+^	Endolysosomes	Voltage-gated, NAADP, PIP_2_	Phagocytosis (Davis et al., 2020), endolysosomal osmo-/mechanosensation, volume regulation (Chen et al., 2021)
Transient Receptor Potential (TRP) (See Table 3 for individual channel types)	Cations	Plasma membrane, endolysosomes, phagosomes	PLC, Ca^2+^, H_2_0_2_, ATP, pH, osmotic cell swelling, exocytosis and other	Survival, polarization, mechanosensing, migration, inflammasome activation, phagocytosis, cytokine production (Santoni et al., 2015) (Santoni et al., 2018) (Dutta et al., 2020)
P2X receptor (P2X4 and P2X7)	Na^+^, K^+^, Ca^2+^	Plasma membrane	ATP	Inflammasome activation, cytokine production (di Virgilio et al., 2018)
Nicotinic Acetylcholine (nACh) receptor (α7 and α4β2)	Na^+^, K^+^, Ca^2+^	Plasma membrane	Acetylcholine	Cytokine production, inflammatory reflex (Báez-Pagán et al., 2015) (Borovikova et al., 2000) (Wang et al., 2002)
5-HT3 (5-Hydroxytryptamine 3) receptor	Na^+^, K^+^, Ca^2+^	Plasma membrane?	Serotonin	Cytokine production (Maehara et al., 2015)
Gamma-Aminobutyric Acid (GABA) type A receptor	Cl^−^, HCO_3_^−^	Plasma membrane	GABA	Autophagy, phagocytosis, cytokine production (Kim et al., 2018) (Shiratsuchi et al., 2009)
Proton	H^+^	Plasma membrane	Voltage-gated	Respiratory burst, intracellular acidosis (DeCoursey, 2010) (Ramsey et al., 2009)
Voltage-Dependent Anion Channel (VDAC)	Anion-selective	Mitochondrial membrane, phagosomal membrane	Voltage-gated	Mitochondrial function (Ramond et al., 2019), bacterial lipid translocation (Danelishvili et al., 2017)
Volume-Regulated Anion Channel (VRAC)	Cl^−^	Plasma membrane	Volume-regulated	Hypotonicity-induced inflammasome activation (Green et al., 2020) Sphingolipid-induced ATP secretion (Burow et al., 2014)
Chloride Intracellular Channel (CLIC) (CLIC1 and CLIC4)	Cl^−^	Cytosol, phagosomal membrane, nucleus	Redox- and pH-sensitive	Phagosomal acidification (Jiang et al., 2012), macrophage deactivation (Malik et al., 2012), inflammasome regulation (Domingo-Fernández et al., 2017)
Transmembrane Protein 16A (TMEM16A)	Cl^−^, HCO_3_^−^	Plasma membrane	Ca^2+^ activated, voltage-gated, PIP_2_	MicroRNA9-induced TGF-β/Smad2 signalling (Zhen et al., 2019; Sui et al., 2020; Bai et al., 2021)
Gap junction hemichannel (Connexins 43 and 37, Pannexin 1)	Cations, anions, large molecules, ATP	Plasma membrane	mechanical stimulation, caspase cleavage, voltage, receptor-induced signalling pathways and other	Migration (Shen et al., 2018), cytokine production, ATP signalling (Rodjakovic et al., 2021) (Adamson and Leitinger, 2014)
Piezo1	Cation-selective	Plasma membrane	Mechanically-activated	Polarization, mechanosensing (Atcha et al., 2021a), bactericidal activity (Geng et al., 2021)

The nomenclature for ion channels can be found in the International Union of Basic and Clinical Pharmacology (IUPHAR)/British Pharmacology Society (BPS) Guide to Pharmacology (IUPHAR/BPS, 2011; Alexander et al., 2011). IP_3_, inositol 1,4,5-trisphosphate; PLC, phospholipase C; ATP, adenosine triphosphate; PIP2, phosphatidylinositol 4,5-bisphosphate; NAADP, nicotinic acid adenine dinucleotide phosphate.

## Membrane potential and macrophages

While the concentrations of ions on each side of a membrane are predominantly determined by transporters, passive movement of ions across the membrane is controlled by ion channels. The principal difference between channels and transporters is that “open” ion channels allow passive diffusion of ions down their electrochemical gradient, whereas transporters depend on the use of energy to move ions, usually, against their gradient (Purves et al., 2001). The membrane potential is a measurement of the difference in electrical potential across a membrane. The gold standard for measuring membrane potential involves the use of electrophysiological methods, however, due to the highly dynamic nature of macrophage plasma membranes, particularly activated macrophages, electrophysiological measurements using patch-clamp electrodes can be a challenge. Nevertheless, several studies, using a variety of electrophysiological methodologies and configurations, have reported the resting plasma membrane potential for non-activated monocytes and macrophages. Gallin and Gallin reported the resting plasma membrane potential of human macrophages as −14.5 ± 5 mV (Gallin and Gallin, 1977), while another study reported the membrane potential of human macrophages as −42 ± 14 mV (Mukherjee et al., 1983). In both these studies cells were isolated from human peripheral blood, although it appears that in the latter study macrophages spent less time in culture. Recordings of the resting plasma membrane potential of macrophages isolated from different species, tissues and cell lines, and using various isolating protocols have also been made (Castranova et al., 1979; dos Reis et al., 1979; Gallin and Livengood, 1980; Gallin, 1981; Ince et al., 1983). The picture that emerges is a high degree of variation between different macrophage populations, and it has been speculated that this may reflect the functional characteristics of the macrophage populations investigated (Mukherjee et al., 1983; Zhang et al., 2010). In line with this idea, recent work using a differentiated macrophage cell line, THP-1 cells, has reported that M2 macrophages have a lower resting plasma membrane potential than M1 macrophages (Li et al., 2016). The significance of the membrane potential is that it impacts certain biological processes, e.g. the driving force for Ca^2+^ entry into a cell when channels selective for this ion are open and membrane lipid dynamics (Zhou X. et al., 2015). Therefore, membrane potential can influence the activation thresholds, efficiency and magnitude of cellular responses (Kadir et al., 2018), and could have significant effects on macrophage activity/function regulating cell signalling molecules and cascades located within or in close proximity to the membrane.

**FIGURE 1:**
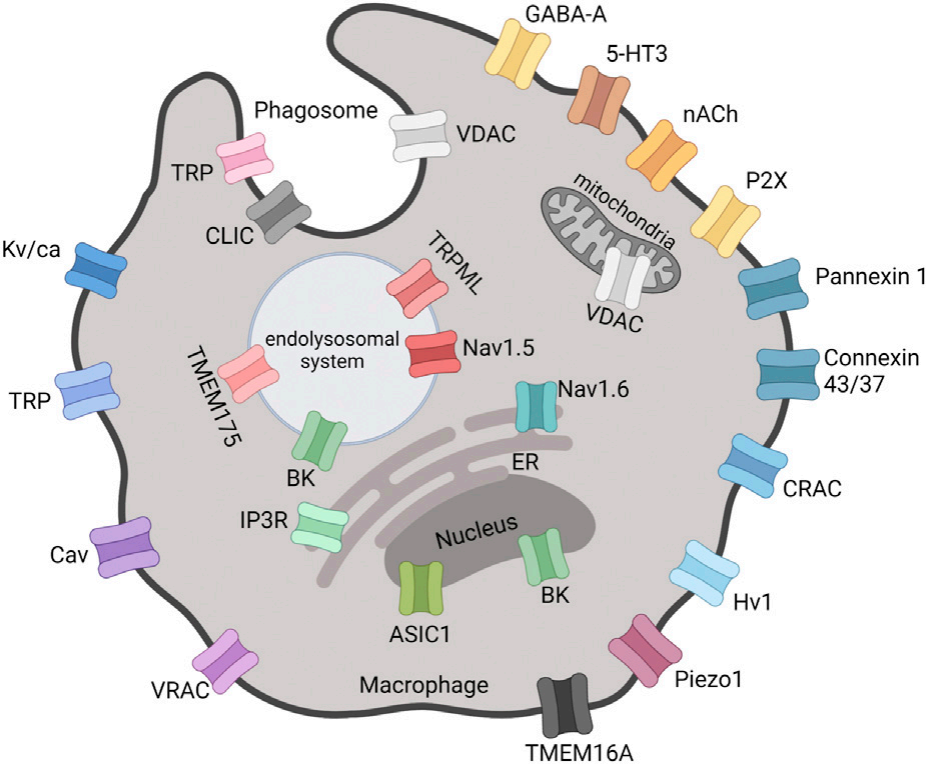
Locations of functional ion channels reported in macrophages. Mitochondria and nucleus are double-membrane-bounded organelles with ion channels located in the inner and/or outer membranes. The outer nuclear membrane is continuous with the ER. VDAC, Voltage-Dependent Anion Channel; GABA-A, Gamma-Aminobutyric Acid type A receptor; 5-HT3, 5-Hydroxytryptamine3 receptor; nACh, Nicotinic Acetylcholine receptor; CRAC, Ca^2+^ Release-Activated Channel; H_v_, Proton voltage-gated channel; VRAC, Volume-Regulated Anion Channel; Ca_v_, Ca^2+^ voltage-gated channel; TRP, Transient Receptor Potential; K_v/Ca_, K^+^ voltage-gated and/or Ca^2+^-activated channel; BK, Big K^+^ channel; Na_v_, Na^+^ voltage-gated channel; TMEM, Transmembrane Protein; IP3R, Inositol 1,4,5-trisphosphate receptor; ER, Endoplasmic Reticulum; ASIC, Acid-Sensing Ion Channel; CLIC, Cl^−^ Intracellular Channel. Created with BioRender.com.

In addition to plasma membrane, intracellular membrane potentials can also regulate cellular and organelle functions, an example being the role of the proton channel Hv1 in the regulation of NADPH oxidase-2 (NOX2) activity in granulocytes (DeCoursey, 2016). In macrophages, mitochondrial membrane potential is known to regulate cellular functions (Wang et al., 2021), and it is expected that the membrane potential of other organelles (Figure 1) will have regulatory roles in macrophages too (Selezneva et al., 2021). However, the majority of research on ion channels in macrophages is focused on the plasma membrane.

It is evident that ion channels regulate ion flux and membrane potential and therefore signalling events at or local to membranes. These signalling outcomes will be dependent on the type of ion channel(s) being activated. Therefore, starting with potassium channels, in the next section we will review the research linking members of different ion channel families with signalling and activation states of macrophages.

## Potassium (K^+^) ion channels

K^+^ channels are the most widely distributed of all the ion channels and are classified into families and subfamilies based on their structure and properties (Alexander et al., 2011). The main function of membrane K^+^ channels is to control the membrane potential. In general, it appears that monocyte-macrophage differentiation and macrophage activation increase the diversity of K^+^ channels expressed by the cell (Vicente et al., 2003; Yoshida, 2017).

Ca^2+^ is a key second messenger in cell signalling with concentration, spatial and temporal parameters all contributing to information conveyed by this divalent cation. K^+^ channels are important in responding to and regulating Ca^2+^ fluxes in cells. For example, extracellular ATP activation of P2Y receptors results in intracellular Ca^2+^ oscillations and promotes IL-6 transcription in macrophages (Hanley et al., 2004). Ca^2+^ -activated K^+^ channels contribute to the intermediate signalling pathways associated with this event by mediating membrane potential oscillations (Hanley et al., 2004). A tight coupling between the Ca^2+^-activated K^+^ channel, K_Ca_3.1, and store-operated Ca^2+^ entry (SOCE) is reported to exist in macrophages. Activated by Ca^2+^ influx through Ca^2+^ release-activated Ca^2+^ (CRAC) channels, K_Ca_3.1 amplifies CRAC current, prolongs Ca^2+^ signalling and Ca^2+^ store refilling (Gao et al., 2010). Macrophages infiltrating into inflamed tissues (Strøbæk et al., 2013), atherosclerotic lesions (Toyama et al., 2008) and transplanted organs have all been shown to express elevated levels of K_Ca_3.1. Toyama and others demonstrated K_Ca_3.1 inhibitors, TRAM-34 and clotrimazole, to significantly reduce macrophage infiltration into atherosclerotic plaques and consequently reduce atherosclerosis development in blood vessels (Toyama et al., 2008). Similarly, Xu and others showed that blocking K_Ca_3.1 shifts macrophage polarization towards M2 macrophage phenotype and stabilises atherosclerotic plaques reducing rupture (Xu et al., 2017). Besides atherosclerosis, Chung and others showed that voltage- and Ca^2+^- activated K^+^ ion channels are capable of influencing macrophage migration by altering the cell volume and shape with implications for neurodegenerative disorders such as HIV1-associated dementia (Chung et al., 2002). Voltage- and Ca^2+^- activated K^+^ channel blockers, charybodotoxin, margatoxin, agatoxin and apamin, were reported to inhibit macrophage migration in the brain, therefore, possibly affecting the levels of neuronal damage (Chung et al., 2002). Taken together, K_Ca_3.1 channels seem to have potential to modulate the intracellular signalling responses of macrophages and contribute to M1/M2 macrophage polarization.

Following several years of controversies about the expression of voltage-gated potassium channels in macrophages (Mackenzie et al., 2003; Park et al., 2006; Felipe et al., 2010), the work of Felipe and others established the presence of both K_v_1.3 and K_v_1.5 in macrophages and microglia, a type of resident macrophage found in the central nervous system. K_v_1.3 and K_v_1.5 form heterotetrameric functional channels (Vicente et al., 2006; Villalonga et al., 2007; Villalonga et al., 2010), and depending on the composition of K_v_1.3/K_v_1.5 in this tetramer, the ion channels take on biophysical and pharmacological characteristics more closely resembling either K_v_1.3 or K_v_1.5 (Vicente et al., 2006; Vicente et al., 2008; Villalonga et al., 2007; Villalonga et al., 2010; Felipe et al., 2010). This highlights the importance of ion channel subunit composition to channel functionality. K_v_1.3 and K_v_1.5 have distinctive channel kinetics, i.e. conductance, activation and inactivation rates, pharmacology and functions in immune cells (Felipe et al., 2010) (Table 2). In macrophages K_v_1.3 is involved in setting the resting membrane potential (Mackenzie et al., 2003; Felipe et al., 2010). Vicente and others reported the reduced activity of voltage-gated K^+^ channels in macrophages inhibits inducible nitric oxide synthase (iNOS) expression (Vicente et al., 2003). In microglia K_v_1.3 blockers reduce production of proinflammatory factors IL-6, TNFα and the enzyme cyclooxygenase-2, protecting from microglia-mediated brain injury (Peng et al., 2014; Fordyce et al., 2005). One possible hypothesis is that K_v_1.3 is essential for the immune cell physiology, while K_v_1.5 contributes to the fine tuning of immune cell responses.

**TABLE 2:** A summary of potassium (K^+^) ion channels identified in macrophages to date.

Channel	Modulation of activity	Functions in macrophages
K_v_1.3	Voltage-gated	Migration (Chung et al., 2002), proliferation, iNOS expression (Vicente et al., 2003)
K_v_1.5	Voltage-gated	Phagocytosis, migration (Park et al., 2006) (Felipe et al., 2010)
K_v_11.1	Voltage-gated	Integrin-dependent binding of apoptotic cells (Vernon-Wilson et al., 2007; Brown and Dransfield, 2008)
K_Ca_1.1 (BK)	Voltage-gated and Ca^2+^ activated	Intracellular signalling, cytokine release (Yoshida, 2017), transcriptional activity (Selezneva et al., 2021) (Papavlassopoulos et al., 2006), large particle phagocytosis, lysosomal functions (Sun et al., 2020)
K_Ca_3.1	Ca^2+^ activated	Ca^2+^ signalling (Gao et al., 2010), migration (Chung et al., 2002), infiltration of atherosclerotic plaques (Toyama et al., 2008), macrophage polarization (Xu et al., 2017)
K_ir_2.1	Lipid-gated	Intracellular signalling (Tsai et al., 2013), proliferation (Vicente et al., 2003)
K_ATP_ (K_ir_6.1/6.2)	ATP-gated	Polarization (Li et al., 2016), atherosclerotic plaque vulnerability (Ling et al., 2013), microglial reactivity (Ortega et al., 2012)
TWIK2	pH, membrane stretch intracellular signalling pathways	Inflammasome activation (Wu et al., 2021) (Di et al., 2018)
Transmembrane Protein 175 (TMEM175)	Conformational changes in Akt kinase	Autophagosome-lysosome fusion (Cang et al., 2015)

ATP, adenosine triphosphate.

Inwardly-rectifying potassium channels allow K^+^ to more easily enter the cell than exit and play a role in setting the membrane potential and are denoted by K_ir_. The inwardly-rectifying K_ir_2.1 was reported to be the target of memantine’s anti-inflammatory effects (Tsai et al., 2013), influence macrophage functional activity and was down-regulated by LPS (Vicente et al., 2003). ATP-sensitive potassium channels (K_ATP_) are also expressed in macrophages are composed of four pore-forming inwardly rectifying K_ir_6.1/K_ir_6.2 subunits and four regulatory sulfonylurea receptor subunits. K_ATP_ channels in macrophages have been seen to modulate cell membrane potential properties, with blockers of the channels promoting a M2 phenotype (Ling et al., 2013; Li et al., 2016), as well as regulate microglial activity (Ortega et al., 2012). A two-pore domain K^+^ channel (TWIK2), which can be activated by a number of chemical and physical parameters including pH, oxygen tensions and stretch, was identified as an essential upstream mechanism for the ATP-induced NLRP3 inflammasome activation (Wu et al., 2021; Di et al., 2018).

Research on Ca^2+^-activated K^+^ channel in macrophages has also shed light on the role of ion channels in non-plasma membrane locations. The large conductance Ca^2+^- and voltage-activated K^+^ channel, K_Ca_1.1, also known as BK channel, has been reported to be present in both plasma and nuclear membranes in macrophages (Yoshida, 2017; Selezneva et al., 2021). Plasma membrane BK channels were found to regulate a disintegrin and metalloprotease domain 17 (ADAM17) enzyme and, hence, the release of TNFα and IL-6 receptor-α from activated macrophages (Yoshida, 2017), while previous studies had reported BK to be central in the NF-κB dependent inflammatory response of macrophages to bacterial LPS (Papavlassopoulos et al., 2006). Interestingly, BK channels appear to be predominantly intracellular in resting macrophages and appear upregulated on the plasma membrane upon LPS stimulation (Yoshida, 2017). BK channels located on the nuclear envelope (NE) were shown to regulate cAMP response element binding protein (CREB) phosphorylation in macrophages (Selezneva et al., 2021). Moreover, this study reported that nuclear Ca^2+^ and calmodulin dependent kinases II and IV were involved in regulation of CREB by nuclear BK channels in macrophages (Selezneva et al., 2021). Similar findings were previously reported in hippocampal neurones (Li et al., 2014). BK channels have been also found on the NE of microglia and were proposed to be involved in microglial activation, facilitating NO and cytokine production, potentially by regulating Ca^2+^ and K^+^ fluxes in the nucleus (Yang et al., 2019). A recent study reported lysosomal BK channels in macrophages to be functionally coupled with transient receptor potential (TRP)ML, discussed below, and regulate large particle phagocytosis through modulating lysosomal exocytosis (Sun et al., 2020). This study highlights an important phenomenon of ion channel coordination to facilitate cell signalling events. Finally, deficiency of transmembrane protein 175 (TMEM175), a K^+^ channel associated with the endolysosomal system has also been shown to result in accelerated autophagosome-lysosome fusion in macrophages (Cang et al., 2015).

## Sodium (Na^+^) ion channels

The recognised role of Na^+^ channels in cell physiology is the propagation of an action potential and depolarization of the plasma membrane. It would, therefore, appear that these channels would not play a role in macrophage biology, see below Electrical Excitability section. However, seven of the nine voltage-gated Na^+^ channel (Na_v_) family members (Na_v_1.1, Na_v_1.3–1.7, and Na_v_1.9) (Carrithers et al., 2007; Sun et al., 2019) have been detected in macrophages, with many of them being associated with intracellular membranes. Na_v_1.5 is expressed in late endosomes and phagolysosomes of macrophages and is reported to regulate phagocytosis and endosomal acidification through triggering Na^+^ efflux and intra-endosomal pH reduction (Carrithers et al., 2007). A later study showed Na_v_1.5 to regulate mycobacteria phagocytosis and phagosome maturation through organelle polarization and localized Ca^2+^ oscillations (Carrithers et al., 2011). Another Na^+^ channel, Na_v_1.6, has also been reported to regulate phagocytosis in macrophages and microglia (Craner et al., 2005), and it localizes to cytoskeletal filaments and endoplasmic reticulum (Carrithers et al., 2007). Several studies have also suggested a role for Na_v_-mediated regulation of macrophage phagocytosis in inflammatory disease progression including myelin degradation in multiple sclerosis (Black et al., 2013), experimental autoimmune encephalomyelitis (Craner et al., 2005) and atherosclerotic lesion development (Sun et al., 2019). Neosaxitoxin, a local anaesthetic and at nanomolar concentrations a selective inhibitor of six Na_v_ isoforms (Na_v_1.1–1.4, 1.6, and 1.7) (Walker et al., 2012), has also been shown to inhibit LPS-induced release of NO produced by iNOS, TNFα and IL-1β from macrophages, thus indicating a role for Na_v_ in inflammatory mediator release from macrophages (Montero et al., 2020).

Decrease in pH has long been associated with acute and inflammatory diseases (Kellum et al., 2004). The Na^+^-permeable channels, acid-sensing ion channels (ASICs), ASIC1, ASIC2a, and ASIC3, have also been identified in macrophages, with ASIC1 immunolocalising to the nucleus, and ASIC2a and ASIC3 being mostly found throughout cell cytoplasm (Ni et al., 2018). Of significance, extracellular acidosis and ASIC activation were found to promote migration, endocytosis, cytokine production and apoptosis in macrophages (Ni et al., 2018; Kong et al., 2013; Foster et al., 2021). It is rather fitting that some classical physiological changes associated with inflammation, such as heat and low pH, may be important sensory cues for macrophages, see the following sections.

## Calcium (Ca^2+^) ion channels

As would be predicted, intracellular Ca^2+^ regulates a variety of functions in macrophages, ranging from cytokine production to phagocytosis (Vaeth et al., 2015). CRAC channels are highly selective for Ca^2+^ compared to other cations and are activated by depletion of ER Ca^2+^ stores (Shim et al., 2015). Briefly, depletion of ER Ca^2+^ stores leads to conformational changes in stromal interaction molecules (STIM) 1 and 2 located on the ER. These conformational changes allow STIM 1 and 2 to oligomerize and form clusters “puncta”, into which CRAC is recruited, resulting in SOCE. ORAI1 is the gene encoding the CRAC channel protein, but the two terms are often used interchangeably. Chauhan et al. reported ORAI1/CRAC to mediate the basal Ca^2+^ influx in macrophages (Chauhan et al., 2018). A tight coupling between CRAC channels, SOCE, and K_Ca_3.1 channels is thought to exist in macrophages, where Ca^2+^-activated K_Ca_3.1 channels amplify the CRAC current, prolonging Ca^2+^ signalling and store repletion (Gao et al., 2010). Loss-of-function mutations in ORAI1 and STIM1 genes abolish CRAC channel function and SOCE in immune cells, resulting in CRAC channelopathies (Shaw and Feske, 2012). However, controversies regarding CRAC/SOCE and macrophage functions exist. STIM1 was reported to be essential for the FcγR-mediated Ca^2+^ entry, phagocytosis, inflammatory cytokine production and autoimmune inflammation (Braun et al., 2009). Another study identified CRAC channels as a principal link between chemokine receptor stimulation and the activation of focal adhesion-related tyrosine kinase Pyk2 and MAPK pathways leading to inflammatory mediator secretion in macrophages (del Corno et al., 2001). Loss of Ca^2+^ entry via CRAC/TRPC1 was found to induce ER stress and apoptosis in macrophages (Conceicao et al., 2019). However, other studies have questioned the importance of Ca^2+^ (Hishikawa et al., 1991; Greenberg et al., 1991; McNeil et al., 1986) and SOCE in macrophage function (Vaeth et al., 2015). Recently it has been shown that M1 and M2 macrophages exhibit different Ca^2+^ entry mechanisms, with M2 macrophages being associated with ORAI1, and TRPC1 channels being associated with M1 macrophages (Nascimento Da Conceicao et al., 2021). Loss of either TRPC1 or ORAI1 limited the functionality of M1 and M2 macrophages respectively. Three other TRP family channels, TRPA1, TRPM7, and TRPP2, have also been reported to mediate the non-ORAI1 component of SOCE in macrophages (Liu et al., 2021). This again highlights the important interplay between ion channels and macrophage functions.

In addition to already discussed CRAC channels, L-type voltage-gated Ca^2+^ channels have been identified in macrophages and suggested to modulate macrophage responses to infection, with roles in reactive oxygen species generation, survival and autophagy (Antony et al., 2015; Sharma et al., 2016). Inositol 1,4,5-trisphosphate receptors, IP3Rs, are ubiquitous Ca^2+^ channels mediating Ca^2+^ release primarily from endoplasmic reticulum stores, have also been implicated in macrophage Ca^2+^ signalling, polarization (Liu et al., 2019) and cytokine production (Staats et al., 2016). However, how many of these functions are dependent on SOCE activation will require further research (Sampieri et al., 2018). Finally, Ca^2+^-permeable two-pore channels, TPCs, found on membranes of the endolysosomal system, together with TRPML channels (see below), have been found to regulate endolysosomal osmo-/mechano-sensation and volume regulation (Chen et al., 2021) and drive macrophage phagocytosis (Davis et al., 2020). TRP channels also account for a significant part of research concerning Ca^2+^-permeable ion channels in macrophages, although they can show varying degrees of selectivity for different cations (Table 3).

**TABLE 3:** A summary of Transient Receptor Potential (TRP) ion channels identified in macrophages to date.

Channel	Permeability	Modulation of activity	Functions in macrophages
TRPA1	Cations	PLC, DAG, cannabinoids, pungent compounds	Cytokine production (Kun et al., 2014), polarization (Wang et al., 2020)
TRPC1	Cations	PLC, Ca^2+^ store depletion, mechanical stretch, conformational coupling	Cytokine production (Zhou et al., 2015a) (Py et al., 2014), polarization (Chauhan et al., 2018)
TRPC3	Cations	Ca^2+^ store depletion, DAG, exocytosis, conformational coupling	Survival (Tano et al., 2014), polarization (Solanki et al., 2014) (Vazquez et al., 2016)
TRPC6	Cations (Ca^2+^>Na^+^)	DAG, PIP_3_, conformational coupling	Phagocytosis (Riazanski et al., 2015)
TRPM2	Cations (Ca^2+^ ≈ Mg^2+^ ≈ Ba^2+^)	ADPR, cADPR, ROS, arachidonic acid, H_2_O_2_, Ca^2+^	Cytokine production (Yamamoto et al., 2008), polarization (Beceiro et al., 2016), inflammasome activation (Zhong et al., 2013)
TRPM4	Monovalent cation selective	Ca^2+^, voltage modulated, PIP_2_	Survival, phagocytosis (Serafini et al., 2012)
TRPM7	Divalent cation selective (Ca^2+^ ≈ Mg^2+^ > Ba^2+^)	Mg^2+^, PIP_2_, extracellular pH, ATP	Polarization (Schilling et al., 2014), regulatory volume decrease (Numata et al., 2007) (Penner and Fleig, 2007)
TRPM8	Cations (Ba^2+^ > Ca^2+^ > Mg^2+^)	PIP_2_, extracellular pH, icilin	Polarization (Khalil et al., 2016)
TRPML1	Cations	PIP_2_, luminal pH	Phagocytosis (Samie et al., 2013)
TRPML2	Cations	PIP_2_, luminal pH	Migration, chemokine release (Sun et al., 2015) (Plesch et al., 2018), endolysosomal viral trafficking (Santoni et al., 2020) (Chen et al., 2020)
TRPML3	Cations (Ca^2+^ > Sr^2+^ > Ba^2+^)	PIP_2_, luminal pH	Membrane trafficking, autophagy (Kim et al., 2009)
TRPP2	Cations	Ca^2+^, translocation, PIP_2_	Store-operated Ca^2+^ entry (Liu et al., 2021)
TRPV1	Cations (Ca^2+^ > Mg^2+^)	heat, protons, PIP_2_	Polarization (Lv et al., 2021), cytokine production (Ninomiya et al., 2017; Sanjai Kumar et al., 2021)
TRPV2	Cations (Ca^2+^ > Mg^2+^)	FcγR signalling, osmotic cell swelling, exocytosis, heat	Migration, phagocytosis (Link et al., 2010) (Nagasawa et al., 2007), cytokine production (Yamashiro et al., 2010), regulatory volume decrease (Muraki et al., 2003)
TRPV4	Cations (Ca^2+^ ≈ Sr^2+^ > Ba^2+^)	Arachidonic acid, 5′6′-EET, osmotic cell swelling, exocytosis	Mechanosensing, polarization (Dutta et al., 2020), phagocytosis, cytokine production (Scheraga et al., 2016)

PLC, phospholipase C; DAG, diacylglycerol; PIP_3_, phosphatidylinositol (3,4,5)-trisphosphate; PIP_2_, phosphatidylinositol 4,5-bisphosphate; (c)ADPR (cyclic) adenosine diphosphate ribose; ROS, reactive oxygen species; ATP, adenosine triphosphate. TRP channel families are; A, ankyrin; C, canonical; M, melastatin; ML, mucolipin; N, no mechanoreceptor potential C; P, polycystic; S, soromelastatin; V, vanilloid; VL, vanilloid-like.

## Transient receptor potential (TRP) ion channels

TRP channels form a large superfamily of cation channels which are important cell sensors. Split into nine families; A, C, M, ML, N, P, S, V, and VL; these can be further divided into subfamilies and display diversity in their permeability to different cations and responses to different chemical and physical stimuli, see Table 3, (Venkatachalam and Montell, 2007; Bouron et al., 2015; Feng et al., 2014). When considering the variety of cellular functions macrophages have, and the dynamic tissue environments in which they perform these functions, it is not surprising that a number of TRP subfamilies have been demonstrated to play regulatory roles in macrophages (Table 3) (Link et al., 2010; Zhong et al., 2013; Schilling et al., 2014; Solanki et al., 2014; Zhou X. et al., 2015; Sun et al., 2015; Khalil et al., 2016; Chauhan et al., 2018; Wang et al., 2020).

Taking the TRP family in alphabetical order, TRPA1 channel was reported to regulate the expression of pro-inflammatory neuropeptides, cytokines and chemokines, exert a mucosal protection in colitis (Kun et al., 2014) and reduce atherosclerosis progression by suppressing macrophage M1 polarization (Wang et al., 2020). Similarly, a recent study showed that ablation of TRPA1 exacerbates M1 macrophage infiltration and renal inflammation after renal ischemia-reperfusion injury in mice (Ma and Wang, 2021).

TRPC1 was found to be crucial for the proinflammatory responses to bacterial infections through stimulating protein kinase C *α* pathway, NF-κB/Jun kinase nuclear translocation and cytokine release (Zhou Y. et al., 2015) (Py et al., 2014). TRPC3 also contributes to macrophage M1 polarization (Solanki et al., 2014; Vazquez et al., 2016; Chauhan et al., 2018) and appears to be an obligatory component of survival mechanisms in human and murine macrophages (Tano et al., 2014). TRPC6 was suggested to have roles in phagocytosis and restore microbicidal function in compromised alveolar macrophages from cystic fibrosis patients (Riazanski et al., 2015).

In the melastatin family of TRP channels plasma membrane TRPM2 was shown to control chemokine production in monocytes, such as macrophage inflammatory protein-2 production (CXCL2) (Yamamoto et al., 2008; Zong et al., 2022). Zhong et al. identified TRPM2 as a key factor linking oxidative stress and NLRP3 inflammasome activation (Zhong et al., 2013). In this study TRPM2 deficient macrophages displayed impaired NLRP3 inflammasome activation and subsequently IL-1β secretion (Zhong et al., 2013). A recent study identified a TRPM2–CD36 axis in macrophages as a critical molecular mechanism underlying atherogenesis (Zong et al., 2022). However, there is some controversy whether TRPM2 promotes inflammation or protects against it. While reactive oxygen species activate TRPM2, one study (Di et al., 2012) reported an ability of TRPM2 to inhibit reactive oxygen species production in macrophages and prevent endotoxin-induced lung inflammation. Similarly, another study reported that the absence of TRPM2 results in greater reactive oxygen species and inflammatory mediator production in macrophages, promotes macrophage M1 polarization and increases gastric inflammation (Beceiro et al., 2016). Moving to other members of this TRP family, TRPM4 was reported to regulate the AKT signalling pathway, phagocytic function and macrophage survival in sepsis (Serafini et al., 2012). Both TRPM7 and TRPM8 have been suggested to have roles in macrophage polarization. TRPM7 blockers were reported to prevent macrophage M2 polarization and inhibitory effects of IL-4 and M-CSF on TNFα production (Schilling et al., 2014). TRPM8 in macrophages determines M1 and M2 actions by regulating TNFα and IL-10 production (Khalil et al., 2016).

TRPML channels belong to the mucolipin TRP subfamily and primarily localize to membrane-bounded vesicles along the endocytosis and exocytosis pathways. In the endolysosomal system TRPML2 was established to be primarily expressed in recycling endosomes, whereas TRPML3 and TRPML1 in early endosomes and late endosomes/lysosomes, respectively (Sun et al., 2015; Plesch et al., 2018). A study by Samie et al. reported that TRPML1 knockout and blockade in macrophages causes inhibition of large particle phagocytosis, whereas TRPML1 overexpression and agonists facilitate the process (Samie et al., 2013). The authors suggest a mechanism, where the binding of phagocytosis target by macrophage leads to PIP2-dependent TRPML1 activation and resulting Ca^2+^ release induces lysosomal exocytosis (Samie et al., 2013). Furthermore, TRPML1 together with BK channels was suggested to be involved in the fusion of phagosomes with lysosomes (Dayam et al., 2015; Cao et al., 2015; Sun et al., 2020) and enhance the degradative lysosome function through transcription factor TFEB activation (Gray et al., 2016). Interestingly, Sun and others reported that TRPML2 expression in macrophages significantly increases upon toll-like receptor activation, while the levels of other TRPML subfamily members do not seem to change with macrophage activation (Sun et al., 2015). Sun and others also showed that TRPML2 knockout leads to reduced production of chemokines such as CCL2 and impaired recruitment of peripheral macrophages (Sun et al., 2015). In support of this, a later study reported that TRPML2 agonist stimulates macrophage migration and the release of CCL2 from macrophages (Plesch et al., 2018). Recently, a role for TRPML2 in endolysosomal viral trafficking and viral infection has been suggested (Santoni et al., 2020; Chen et al., 2020). TRPML3 is thought to have a role in the regulation of endocytosis, membrane trafficking and autophagy (Kim et al., 2009). A recent study reported TRPML3 in alveolar macrophages to regulate elastase MMP-12 uptake, influencing chronic obstructive pulmonary disease development (Spix et al., 2022).

Extensive research has been carried out on TRPV1 and its role in pain, and while a number of drugs have entered clinical trials, these drugs are associated with significant side effects which has limited their utility, see Table 4 (Moran, 2018). In macrophages TRPV1 inhibits M1 polarization via Ca^2+^/CaMKII/Nrf2 signalling pathway (Lv et al., 2021) and influences cytokine production in models of osteoarthritis, sepsis (Ninomiya et al., 2017), inflammatory tissue fibrosis (Okada et al., 2011) and viral infection (Sanjai Kumar et al., 2021). Interestingly, a recent study suggested that inflammation-mediated macrophage polarisation induces TRPV1/TRPA1 heteromeric channel formation with key roles in endometriosis pain (Zhu et al., 2022). A second member of TRPV subfamily, TRPV2 channel, plays regulatory roles in a number of macrophage functions including chemotaxis and early phagocytosis (Link et al., 2010; Nagasawa et al., 2007), podosome formation (Nagasawa and Kojima, 2012) and cytokine production (Yamashiro et al., 2010). Moreover, TRPV2 and TRPM7 in macrophages are thought to control regulatory volume decrease (RVD), which leads to NLRP3 inflammasome activation (Santoni et al., 2015; Compan et al., 2012; Liedtke, 2006). During RVD TRPV2 is translocated to the plasma membrane where it induces cellular permeabilization, while TRPM7 acts as a controller of the regulatory volume decrease process (Muraki et al., 2003). TRPM7 blockade was reported to delay the RVD, reduce cell permeabilization and IL-1β release in macrophages (Numata et al., 2007; Penner and Fleig, 2007). An interesting member of TRPV family is TRPV4. It responds to mechanical stimuli and has been shown to regulate macrophage phagocytosis and cytokine secretion (Scheraga et al., 2016). A more recent study by Scheraga R. et al (2020) established that TRPV4 enhances macrophage bacterial clearance and reduces proinflammatory secretion of cytokines through MAPK molecular pathway switching (Scheraga R. et al., 2020). TRPV4 roles in macrophages appear to be important in inflammation and disease progression where mechanical stimuli may be of significance e.g. ventilator/chemically-induced injury (Hamanaka et al., 2010; Balakrishna et al., 2014; Scheraga R. G. et al., 2020) and tuberculosis (Naik et al., 2020). A role for TRPV4 in matrix stiffness-induced macrophage polarization has also been suggested (Dutta et al., 2020). Finally, there are reports that TRPV4 can contribute to the formation of specialised macrophage phenotypes (Goswami et al., 2017, 2021).

**TABLE 4:** Ion channel-targeted drugs in clinical trials I-III or approved.

Ion channel	Drug	Disease	Clinical trial	Profile
K_V_1.3	Dalazatide (Shk-186). Selective blocker	Psoriasis	Phase Ib	Moderate effect on plaque size, attributed to T cell effects (Tarcha et al., 2017)
K_V_1.5	XEN-D0103 (S66913). Selective blocker	Healthy volunteers	Phase I	Mechanistic proof of concept. No significant side-effects. (Ford et al., 2016)
		Paroxysmal atrial fibrillation	Phase II	Well tolerated, no significant clinical benefit (Shunmugam et al., 2018; Camm et al., 2019)
K_Ca_1.1	MaxiPost (BMS-204352). Channel opener	Healthy volunteers	Phase I	Well tolerated (Jensen, 2002)
		Stroke	Phase Ib-II	Well tolerated in stroke patients (Jensen, 2002)
		Stroke	Phase III	Failed to show greater clinical efficacy than placebo (Jensen, 2002)
		Healthy volunteers	Phase I	Mechanistic proof of concept. Dilation of extra and intracerebral arteries. Caused headache (Al-Karagholi et al., 2020)
	VSN16R, Channel opener	Healthy volunteers	Phase I	Initially designed as cannabinoid, developed to control neuronal excitability and spasticity in MS, subsequently identified as channel opener. Well tolerated. (Baker et al., 2017)
		Multiple sclerosis	Phase II	Failed to show decrease in spasticity compared to control. May be related to short half-life (ClinicalTrials.gov, NCT02542787, 2018)
	GAL-021, Channel blocker	Healthy volunteers	Phase I	Mechanistic proof of concept. Well tolerated. Stimulated ventilation (McLeod et al., 2014)
		Opioid-induced respiratory depression	Phase II	Respiratory stimulatory effect. No safety issues reported (Roozekrans et al., 2014)
K_Ca_3.1	Senicapoc (ICA-17043). Channel blocker	Sickle cell disease	Phase I	Well tolerated (Ataga et al., 2006)
		Sickle cell disease	Phase II	Significantly increased haemoglobin levels compared to placebo (primary end point). Well tolerated and safe (Valle and Lemberg, 1991)
		Sickle cell disease	Phase III	Improvements in anaemia and haemolysis in the treatment group, was not reflected in improvements in the rate of pain crises (primary endpoint) compared to Placebo. Increased rate of urinary infection in treatment group (Ataga et al., 2011, 2021)
		COVID-19 severe respiratory insufficiency	Phase II, open label	Treatment significantly lowered PaO_2_/FiO_2_ ratio (Granfeldt et al., 2022)
K_ATP_	Glibenclamide, Inhibits the channel regulatory subunit SUR1	Type 2 diabetes	Approved	Antidiabetic treatments can cause GI effects, nausea, bloating, weight gain. (Beltrand et al., 2019) SUR1 regulatory subunit also interacts with TRPM4, with this interaction being suggested as a therapeutic target in stroke (Koivisto et al., 2022)
	Levcromakalim (Cromakalim). Channel opener	Healthy volunteers	Phase I-II	Proof of concept. Arterio-selective vasodilation. (Longman and Hamilton, 1992)
		Nocturnal asthma	Phase Ib	Treatment reduced early morning fall in FEV1 seen in asthma patients compared to placebo. (Williams et al., 1990)
	BRL-38227 (active enantiomer of Cromakalim). Channel opener	Asthma	Phase II	No treatment effect on baseline FEV1 or after histamine or methacholine challenge in asthma patients Reports of increased headache (K_ATP_ channel may contribute to the pathophysiology of headache and migraine) (Kidney et al., 1993; Al-Karagholi et al., 2017)
Na_V_1.7	Vixotrigine, Funapide, PF-05089771, AZD-3161, GD-0276, GDC-0310. Channel blocker	Pain	Phase I and II	Numerous clinical trials investigating Na_v_1.7 blockers in pain conditions. (Alsaloum et al., 2020; Kushnarev et al., 2020)
CRAC	RP3128. Channel modulator	Healthy volunteers	Phase I	Safe and well tolerated. Treatment reduced baseline plasma levels of TNFα and IL-4 (Barde et al., 2021)
	Auxora (CM4620), Orai1 inhibitor	Acute pancreatitis	Phase II, open label	Patients had acute pancreatitis, systemic inflammatory response syndrome and hypoxemia. Small sample size, insufficient statistical power (Bruen et al., 2021)
		Severe COVID-19 pneumonia	Phase II	Addition of Auxora to treatment regieme of patients on standard therapeutic protocols including glucocorticoids. Secondary endpoint (all-cause mortality Day 30) significant decrease compared to control. Other parameters trending towards positive effect of drug. Well tolerated. (Bruen et al., 2022)
L-type calcium channel	Channel blockers include, diltiazem verapamil, nifedipine	Hypertension, myocardial ischemia	Approved	L-type calcium channels consist of Ca_V_1.1-Ca_V_1.4 depending on the alpha subunit. Selectivity of blockers is dependent on subunit compositions. (Zamponi et al., 2015)
TRPA1	GC17536. Receptor antagonist	Diabetic neuropathic pain	Phase II	Proof of concept. Study demonstrated a significant effect on pain scores by the antagonist in sub-group of patients. PK and bioavailability issues have prevented further development of compound. (Fernández-Carvajal et al., 2020; Koivisto et al., 2022)
TRPM8	(1R,2S,5R)-N-(2-(2-pyridinyl)ethyl)-2-ispropyl-5-methylcyclohexancarboxamide + menthoxypropanediol. TRPM8 agonists	Pruritus	Phase II	Proof of concept. Topically applied agonist significantly reduced pruritus and secondary endpoints compared to vehicle control (Ständer et al., 2017)
	Cryosim-3 (1-diisopropylphosphorylnonane). TRPM8 agonist	Dry eye	Phase II	Proof of concept. Wipe on solution of agonist significantly improved symptoms of dry eye after 1- and 2-weeks application (Yang et al., 2017)
	PF-05105679, TRPM8 channel blocker	Healthy volunteers	Phase I	Inhibition of pain in cold test assay. Increased feeling of hotness reported as an adverse effect (Winchester et al., 2014)
TRPV1	Capsaicin, Agonist (topical application)	Pain	Approved	Works via channel desensitization, clinical effect in osteoarthritis, post-herpetic neuralgia, diabetic polyneuropathy (Fattori et al., 2016; Moran and Szallasi, 2018; Koivisto et al., 2022)
	Resiniferatoxin, Potent agonist	Osteoarthritis pain	Phase Ib	Placebo controlled open labelled. Moderate pain on injection which resolved within 24 h. Currently considered as a molecular scalpel to ablate sensory neurons (Moran and Szallasi, 2018)
	AZD-1386, AMG517, SB-705498, JNJ-39439335, PAC-14028, etc. TRPV1 antagonists	Pain	Phase I-II	First generation TRPV1 antagonists caused transient increase in body temperature and decrease in noxious heat sensation. Second generation drugs are attempting to overcome these limitations. AZD-1386 antagonist did not show efficacy in osteoarthritis pain (Miller et al., 2014; Moran & Szallasi, 2018; Fernández-Carvajal et al., 2020)
	SB-705498. TRPV1 antagonist	Refractory chronic cough	Phase II	Antagonist decreased cough response to capsaicin challenge at 2 h compared to placebo, no effect on objective cough frequency over 24 h. Well tolerated (ClinicalTrials.gov, NCT01476098, 2016)
		Seasonal allergic rhinitis	Phase II	No effect of antagonist compared to placebo (Bareille et al., 2013)
	Asivatrep (PAC-14028). TRPV1 antogonist	Atopic dermatitis	Phase II-III	Topical application of antagonist improved primary and secondary end points of dermatitis compared to vehicle group. Well tolerated (Lee et al., 2019; Park et al., 2022)
TRPV4	GSK2798745, Channel blocker	Healthy volunteers and heart failure patients with lung congestion	Phase I	PK and safety evaluation. Drug was found to be well tolerated in healthy subjects and patients with lung congestion in heart failure (Goyal et al., 2019; Stewart et al., 2020)
P2X7	AZD9056, Receptor antagonist	Rheumatoid arthritis	Phase II	Antagonist administered with DMARD showed no significant enhanced efficacy compared to placebo. Antagonist was well tolerated (Keystone et al., 2012)
		Crohn’s disease	Phase II	Remission rate of disease was significantly higher in the treatment group compared to placebo, with a marked decrease in some metrics of Crohn’s disease. Drug was well tolerated (Eser et al., 2015)
	CE-224,535, Receptor antagonist	Rheumatoid arthritis	Phase II	Trial was carried out in RA patients who were inadequately controlled by the DMARD methotrexate. Antagonist was no more efficacious than control and had comparable safety profile (Stock et al., 2012)
	JNJ-54175446, Receptor antagonist	Healthy volunteers	Phase I	Evaluation of PK and safety of brain barrier-permeable receptor antagonist. Drug was well tolerated (Timmers et al., 2018)
α7-nAChR	GTS-21 (DMXB-A), α7 full agonist, binds α4β7	Healthy volunteers	Phase I	Drug was well tolerated, suggested improvement in cognitive ability compared to placebo (Kitagawa et al., 2003)
		Schizophrenia	Phase II	Significant neurocognitive improvement compared to placebo. Well tolerated, 1 patient had decreased white blood cell count (Olincy et al., 2006; Freedman et al., 2008)
	Encenicline (EVP-6124), α7 full agonist	Schizophrenia	Phase I-II	Improvement in impaired cognitive ability in Schizophrenia with drug compared to control. Trials currently on hold due to rare adverse reactions seen with the drug (Keefe et al., 2015)
	Nelonicline (ABT-126), α7 full agonist	Alzheimer’s disease	Phase II	Well tolerated, no significant improvement in cognitive ability compared to placebo (Gault et al., 2015; Florian et al., 2016)
		Schizophrenia	Phase I-II	Significant cognitive effect in non-smokers compared to placebo, however, not repeated in second study (Haig et al., 2016b; 2016a)
	AVL-3288, α7 allosteric modulator	Schizophrenia	Phase I-II	Drug was well tolerated. No evidence of increased efficacy of drug against control. ClinicalTrials.gov, NCT02446340, 2015
				Other partial agonists, TC-5619 and RG3487, also report minimal efficacy in this condition (Gee et al., 2017; Kantrowitz et al., 2020)
α4β2-nAChR	TC-6683 (AZD1446), Full agonist	Attention-deficit/hyperactivity disorder	Phase II	No significant effect of drug on clinical endpoints compared to placebo. Drug was well tolerated (Jucaite et al., 2014)
5-HT_3_ receptor	Dolasetron, Ondansetron, Granisetron, Tropisetron. Receptor antagonists	Emesis	Approved	Individual antagonists are used to treat nausea and vomiting induced by radiotherapy, chemotherapy, surgery and gastroenteritis (Thompson, 2013)
GABA_A_ receptor	Barbiturates: Pentobarbital, Sodium thiopental, Primidone, Positive allosteric modulators of GABA_A_ receptor (high concentrations act as agonists)	Anti-epilepsy, general anaesthesia	Approved	Barbiturates used as anxiolytics and sedatives have been replaced by benzodiazepines (López-Muñoz et al., 2005)
	Benzodiazepine: Diazepam, Lorazepam, Oxazepam, Positive allosteric modulators of GABA_A_ receptor	Anxiolytics, sedatives, anti-convulsant, alcohol withdrawal	Approved	Replaced the barbiturates. Can bind other proteins found in macrophages e.g. TSPO18 (Griffin et al., 2013; Jembrek and Vlainic, 2015)
Connexin 43	Tonabersat (SB-220453). Cx43 hemichannel blocker	Migraine	Phase II	Multiple studies investigating prophylactic effects of drugs. No clear evidence for effectiveness (Cao and Zheng, 2014)
	Alpha connexin carboxyl terminus 1 (αCT1, ACT1). Modulates connexin 43 protein interactions	Wound healing	Phase I-II	Wound healing and/or scar reduction in surgical incision, chronic venous leg ulcers, diabetic foot ulcers (Montgomery et al., 2018)
	Rotigaptide (ZP-123), Connexin 43 modulator peptide	Healthy volunteers	Phase I	Well tolerated. No effect on basal vascular tone, no effect on induced vasodilation (Lang et al., 2008)
	Danegaptide (GAO-134). Connexin 43 modulator peptide	Acute myocardial infarction	Phase II	Did not improve myocardial salvage compared to placebo (Engstrøm et al., 2018)

The table includes a non-exhaustive list of drugs which have entered clinical trials against various ion channels. The drugs show high selectivity for the intended target, or the major therapeutic outcome is believed to be via indicated target. It is presumed that most of the effects reported are due to engaging the intended ion channel in non-macrophage cells. DMARD, disease-modifying antirheumatic drug; FEV_1_, forced expiratory volume in 1 s; MS, multiple sclerosis; RA, rheumatoid arthritis; SUR1, sulfonylurea receptor-1.

Together these studies highlight the importance and complexity of these “sensory” channels in macrophages, and we refer the reader to some in-depth reviews of this channel superfamily (Santoni et al., 2018; Nguyen et al., 2022). However, it is important to discriminate in ion channel knock-down studies if the reported effects are directly as a result of ion channel modulation in the macrophage or via an up-stream mechanism, e.g. ion channel modulation in T lymphocytes can subsequently impact macrophage activity (Okada et al., 2011; Ma and Wang, 2021). With time, more roles for TRP channels in macrophage regulation are being established. Functional modulation of TRP channels in macrophages may represent a rich area for the development of therapies to treat a range of inflammatory diseases.

## Ligand-gated ion channels (LGICs)

By comparison to voltage-gated ion channels, which open to changes in membrane potential, a LGIC opens in response to the binding of certain chemical messengers. Therefore, the opening of a LGIC will not only be determined by its expression but also the concentration of the relevant ligand which has access to the ligand-binding site on LGIC. This is an important caveat when investigating LGICs in macrophages since many of the endogenous ligands for LGICs are not found in tissues at sufficient concentrations to activate the channel and are not traditionally synthesised by cells found in inflammatory lesions. Nevertheless, this does not negate the possibility that synthetic openers, blockers and allosteric modulators of macrophage LGICs may have therapeutic value.

P2X receptors are a family of ATP-gated cation channels comprising seven subtypes, P2X1-7. Although several P2X receptor mRNA transcripts, P2X1, P2X3-5 and P2X7, were detected in macrophages, to date only P2X4 and P2X7 have been reported to have defined functional roles in macrophages, with their activation being associated with the onset of inflammatory reactions (Kessler et al., 2011; Bowler et al., 2003). The stimulation of P2X4, which has approximately 100-fold higher affinity for ATP than P2X7 (Jarvis and Khakh, 2009; Asatryan et al., 2018), in macrophages was found to trigger Ca^2+^ influx and p38 MAPK phosphorylation, causing cytosolic phospholipase A_2_ activation and cyclooxygenase-dependent release of the lipid mediator prostaglandin E_2_ (Ulmann et al., 2010). Another study reported a role for P2X4 in chemokine secretion by macrophages (Layhadi et al., 2018). In microglia P2X4 was reported to trigger the release of brain-derived neurotrophic factor and initiate a core pain signalling pathway (Beggs et al., 2012; Masuda et al., 2014). Many studies agree that P2X7 plays a prominent stimuli-dependent role in controlling inflammasome activation and macrophage function. P2X7 activation was reported to promote the efflux of K^+^, activate the NLRP3 inflammasome and cause release of IL-1β from macrophages (Ferrari et al., 1997; Ferrari et al., 2006; Piccini et al., 2008; Barberà-Cremades et al., 2012; Mariathasan et al., 2006; Muñoz-Planillo et al., 2013; Pétrilli et al., 2007). Moreover, Franceschini et al. showed that P2X7 and NLRP3 inflammasome closely interact and colocalize at discrete subplasmalemmal sites in microglia and macrophages (Franceschini et al., 2015). Absence of the P2X7 was demonstrated to result in an inability of macrophages to release IL-1β from macrophages in response to ATP and, therefore, impaired cytokine signalling cascades (Solle et al., 2001). Hemichannel Pannexin 1 was found to be required for the IL-1β release by the ATP-gated P2X7 (discussed below) (Pelegrin and Surprenant, 2006). There have also been reports of P2X7 receptor involvement in ATP-induced ATP release (Suadicani et al., 2006), microvesicle shedding (MacKenzie et al., 2001) and phagocytosis (Gu et al., 2010).

Nicotinic acetylcholine (nACh) α7 receptors are another ligand-gated cation-selective channel which contributes to shaping macrophage behaviour. Interestingly, these channels expressed in macrophages participate in a so-called anti-inflammatory vagal reflex or cholinergic anti-inflammatory pathway which integrates the nervous and immune system. In this reflex acetylcholine (ACh) has an anti-inflammatory effect on macrophages (Wang et al., 2002). Studies show that nAChα7 receptor activation in macrophages is capable of modulating inflammation by inhibiting NF-κB nuclear translocation (Yoshikawa et al., 2006), activating the JAK2/STAT3 pathway (de Jonge et al., 2005; Joe et al., 2011) and selectively inhibiting pro-inflammatory cytokine production while leaving anti-inflammatory cytokines undisturbed (Báez-Pagán et al., 2015; Borovikova et al., 2000; Wang et al., 2002). Tsoyi et al. have suggested that nAChα7 receptor activation in macrophages can mediate anti-inflammatory effects by activating the PI3K/Akt/Nrf-2 pathway and upregulating heme oxygenase-1 (Tsoyi et al., 2011). Due to the high levels of cholinesterase activity in tissues a valid question to ask is the source of ACh which activates macrophage nACh receptors. Importantly, along with the splenic nerves research now indicates that immune cells act as a source of ACh in the spleen and this contributes to the anti-inflammatory vagal reflex (Fujii et al., 2017), although it must be acknowledged that human nAChα7 receptors require high concentration of ACh (EC50 = 128 ± 12 μM) to be activated (Gill et al., 2011). Other nACh receptor subunits have been reported in macrophages including α4β2 (Kiguchi et al., 2012; Kiguchi et al., 2018), α2, α5 and α10 (Kawashima et al., 2007), however, their functions in macrophages require further investigation.

In addition to the above mentioned nACh and P2X receptors, 5-Hydroxytryptamine 3 (5-HT3) receptor is another LGIC found in macrophages where it is thought to modulate cytokine secretion (Seidel et al., 2008; Maehara et al., 2015). The role of 5-HT (serotonin) in inflammation is historically associated with the vasculature (Majno and Palade, 1961), with the majority of peripheral 5-HT being stored in platelets and released upon platelet activation. However, other possible sources of peripheral serotonin could include lymphocytes, macrophages and mast cells (Herr et al., 2017). Maehara and others suggest a therapeutic potential for 5-HT3 receptor antagonists targeting peritoneal macrophages expressing the receptors in post-operative ileus (Maehara et al., 2015).

The presence of functional Cl^−^-permeable gamma-aminobutyric acid (GABA) type A receptors in macrophages has also recently been reported (Kim et al., 2018). GABA is one of the principal neurotransmitters in the brain and is long associated with GABAergic cells, however, GABA can be synthesised by a number of cells including B lymphocytes (Zhang et al., 2021). Interestingly, GABA has been attributed with both inhibitory effects on phagocyte activation and pro-inflammatory functions (Bhandage and Barragan, 2021). While GABA appears to promote monocyte differentiation into anti-inflammatory macrophages that secrete IL-10 (Zhang et al., 2021), the treatment of macrophages with GABA or GABAergic drugs was shown to promote autophagy activation, enhance phagosomal maturation and antimicrobial responses against mycobacterial infection (Kim et al., 2018). Propofol, a commonly used anaesthetic agent, was reported to bind to GABA_A_ receptors in macrophages and inhibit macrophage phagocytosis, with potential implications for post-surgical wound healing (Shiratsuchi et al., 2009). Furthermore, a recent study highlights a role for GABA_A_ receptors in macrophages in pressure overload-induced heart failure (Bu et al., 2021).

## Further macrophage ion channels

In addition to the members of the large ion channel families a number of other ion channels have been reported to influence macrophage behaviour. Proton voltage-gated channel, H_v_1, is reported to regulate the respiratory burst during phagocytosis, limit membrane depolarization and intracellular acidosis in macrophages (DeCoursey, 2010) (Ramsey et al., 2009). In microglia, H_v_1 was reported to enhance reactive oxygen species generation contributing to brain damage at early time points after ischemic stroke (Wu et al., 2012).

Voltage-Dependent Anion Channels (VDAC), initially described as mitochondria-associated porins (Schein et al., 1976), have been identified on plasma (Bàthori et al., 1999) and phagosomal membranes as well. In addition to the pivotal roles of mitochondria in cellular metabolism (Ramond et al., 2019), in macrophages VDACs appear to play a more negative role during infection, being crucial for bacterial growth and survival within phagosomes. The channels were reported to colocalize and interact with bacterial proteins and aid bacterial lipid translocation (Danelishvili et al., 2017).

Several studies report the presence of functional volume-regulated anion channels, VRACs, in macrophages. Burrow and others suggested VRAC to be a functional link between sphingolipid and purinergic signalling systems in macrophages, with roles in phagocytosis, migration and inflammation (Burow et al., 2014). A recent study by Green et al. reports LRRC8A, a pore-forming subunit of VRAC, to be essential for hypotonicity-induced NLRP3 inflammasome activation in macrophages (Green et al., 2020).

Cl^−^ intracellular channels (CLIC), in particular CLIC1, is a family of ion channels permeable to Cl^−^ ions in macrophages, which was reported to regulate macrophage function through modulation of phagosomal acidification and reactive oxygen species production (Jiang et al., 2012). Additionally, iNOS-induced nuclear translocation of another family member, CLIC4, was reported to be essential for macrophage transition from a pro- to anti-inflammatory state (Malik et al., 2012). Furthermore, both CLIC1 and CLIC4 have been suggested to be involved in the NLRP3 inflammasome regulation (Domingo-Fernández et al., 2017). One more Cl^−^ channel found in macrophages is transmembrane protein 16A, TMEM16A (Shi et al., 2020), which has been reported to mediate microRNA9-induced TGF-β/Smad2 signalling implicated in LPS-induced sepsis (Zhen et al., 2019; Sui et al., 2020; Bai et al., 2021).

A number of studies have pointed to the presence of hemichannels and gap junctions in macrophages (Levy et al., 1976; Porvaznik and MacVittie, 1979; Eugenín et al., 2003; Jara et al., 1995). Plasma membrane hemichannels are one half of gap junction channels which allow direct ion transfer between cytoplasms of contacting cells and thus allow metabolic and electrical synchronization between the contacting cells. Hemichannels Connexin 43 (Eugenín et al., 2003; Jara et al., 1995; Beyer and Steinberg, 1991; Dosch et al., 2019), Connexin 37 (Kwak et al., 2002; Lu et al., 2018) and Pannexin 1 (Chen et al., 2019; Adamson and Leitinger, 2014; Pelegrin and Surprenant, 2006) have been identified in macrophages to date and proposed to affect macrophage activation and a range of functions (Rodjakovic et al., 2021), including macrophage migration (Shen et al., 2018), ATP signalling (Pelegrin and Surprenant, 2006) and cytokine production (Dosch et al., 2019). Importantly, growing evidence suggests that the presence of hemichannels and gap junctions in macrophages is crucial for many physiological and pathophysiological processes, such as heartbeat propagation (Hulsmans et al., 2017), immunomodulatory communication in the lungs (Westphalen et al., 2014) and intercellular communication in intestine (Al-Ghadban et al., 2016).

Finally, a novel mechanically-activated cation channel Piezo1 has been reported in macrophages. Piezo1 roles are proposed to include mechanical stimuli sensing, potentially via Piezo1 and integrins crosstalk and modulation of macrophage polarization (Atcha et al., 2021a; Atcha et al., 2021b), toll-like receptor 4-mediated enhancement of macrophage bactericidal activity (Geng et al., 2021) and macrophage migration (unpublished data). Recently, this channel has also been demonstrated to be a key regulator of erythrocyte phagocytosis by macrophages and iron metabolism (Ma et al., 2021). Since iron metabolism, inflammation and immunity are intimately linked (Ganz and Nemeth, 2015), this area of research deserves further consideration. Piezo1 is considered to be a promising candidate for therapeutic intervention against atherosclerosis and other cardiovascular diseases (Umar Shinge et al., 2022), and it will be interesting if this channel along with other mechano-sensing channels represent a mechanism by which physical stimuli together with chemical stimuli, such as PAMPs and DAMPs, can be integrated.

## Research questions, future prospects and therapeutics

### Methodology

The gold standard technique for identifying functional ion channels in a membrane is patch clamping electrophysiology. Being a common technique used to obtain electrophysiological data from many cell types, it can be technically challenging in cells which have dynamic membranes, as is the case for activated macrophages (Pi et al., 2014). Macrophages are heterogeneous populations of cells, and, since patch clamping experiments are carried out on single cells, investigators need to be aware of sampling issues, i.e. macrophages which are amenable to electrophysiological recordings may not represent bulk phenotypic behaviour of the macrophage population. Interestingly, research in neurons has developed Patch-seq protocols, where electrophysiological and transcriptomic data can be collected from the patched cell (Lipovsek et al., 2021). To the best of our knowledge this technique has not been investigated in macrophages to date, however, this could represent a powerful technique by which macrophage functional phenotypes could be linked to gene expression profiles. Cells have also been patch clamped *in-situ* e.g. neurons in brain slices. It could therefore be possible to patch clamp macrophages in tissue slices, e.g. spleen, granuloma, or even tumours to investigate tumour associated macrophage (TAM) activity. This methodology may also overcome some of the macrophage plasticity issues encountered when investigating these cells *in-vitro*.

One limitation to patch clamping is that it is low throughput, although automation of this technique is possible for some cell types. To investigate ion-channel effects in multiple cells simultaneously *in vitro,* fluorescent dyes can be used, however, these dyes require to be of sufficient spatial and temporal resolution to characterise ion channel dynamics (Haworth and Brackenbury, 2019). Furthermore, ion channels may have functions which are not dependent on their “channel” function. An example of this is the recent demonstration that the role of voltage-gated Ca^2+^ channels in regulating T-lymphocyte function is independent of cation flux (Cahalan, 2022). To investigate this possibility in macrophages a combination of electrophysiological, genomic, proteome and interactome methodologies will be required.

Care must also be taken when using pharmacological and genetic approaches to investigate ion channel functions. Channel blockers/openers must be chosen carefully and their relative selectivity for a particular target should be considered. This is especially important in ion channel research where the selectivity of pharmacological tools is often dependent on the subunit composition and dynamics of the ion channel. Also, it must be recognised that some ion channels can be found at multiple locations within the cell, where they may play different roles in cellular pathways. This confounding factor must be taken into account when using pharmacological and genetic approaches to manipulate ion channel activities and investigate their downstream effects.

### Ion channel coordination

The coordination of different ion channels in a single cell to orchestrate a particular cellular function is well recognised in cell types such as neurons and smooth muscle. Evidence suggests such coordinated behaviour of ion channels is also present in macrophages. One example would be K^+^ channel opening creating a driving force for Ca^2+^ entry into the cells (see above). A number of ion channels, TWIK2, TRP, P2X, VRAC, and CLIC channels, are reported to regulate NLRP3 inflammasome (Li et al., 2021). Ion channels can also regulate cell volume changes which activate NLRP3. TRPV2 and TRPM7 in macrophages are thought to control the RVD (Liedtke, 2006; Compan et al., 2012; Santoni et al., 2015). Moreover, TRPM2-mediated Ca^2+^ influx has been suggested to be important for inflammasome activation (Zhong et al., 2013). Cytosolic K^+^ efflux is a common trigger involved in inflammasome activation. P2X7 activation was reported to promote the efflux of K^+^ and activate NLRP3 in macrophages (Ferrari et al., 1997, 2006; Mariathasan et al., 2006; Piccini et al., 2008; Barberà-Cremades et al., 2012; Muñoz-Planillo et al., 2013) (Pétrilli et al., 2007). Recently, Di et al. identified a K^+^ channel TWIK2 as another mediator of K^+^ efflux and inflammasome activation in macrophages (Wu et al., 2021) (Di et al., 2018). Finally, intracellular Cl^−^ efflux, an event downstream of K^+^ efflux, is also reported to be critical upstream event for inflammasome activation. Two Cl^−^ selective ion channels, VRAC and CLIC, have been reported to participate in inflammasome regulation in macrophages (Green et al., 2020) (Domingo-Fernández et al., 2017). The important point to make is that combinations of ion channels are involved in inflammasome activation; and these combinations will be dependent on the stimuli. Phagocytosis and polarization are other examples of macrophage functions discussed in this review, which are modulated by integrated ion channel networks (Table 1). It would not be surprising if the effects of individual ion channels on macrophage behaviour are in fact dependent on the actions of other channels. If so, the integration of these ion channel “circuits” with traditional cell signalling pathways in macrophages will need to be investigated.

### Electrical excitability

In neurons the different composition of voltage-gated ion channels results in different firing properties (Berger and Crook, 2015) (Drion et al., 2015) but as the name suggests these ion channels are regulated my membrane voltage i.e. neurons are electrically excitable. Macrophage express an array of functional ion channels, some of which are voltage-gated. This leads us to the controversial question, are macrophages electrically excitable? While macrophages are generally considered non-excitable cells, the presence of gap junction channels Connexins in macrophages allows cardiac macrophages to facilitate electrical conduction through the distal atrioventricular node, where conducting cells densely intersperse with elongated macrophages expressing Connexin 43. When coupled to spontaneously beating cardiomyocytes via connexin-43-containing gap junctions, cardiac macrophages have a negative resting membrane potential and depolarize in synchrony with cardiomyocytes (Hulsmans et al., 2017). Moreover, computer simulations to quantitatively assess plausible effects of macrophages on electrotonically coupled cardiomyocytes showed that macrophages can depolarize resting cardiomyocytes, shorten early and prolong late action potential duration, with effects depending on coupling strength and individual macrophage electrophysiological properties, in particular resting membrane potential and presence/absence of K_ir_2.1 channel (Simon-Chica et al., 2021). To study this further, in-situ measurements of electrophysiological properties of macrophages in contact with other cells will have to be performed.

### Therapeutic potential

As indicated in the introduction, macrophages not only have central roles in immune and inflammatory responses but participate in general homeostatic responses in the body (Murray and Wynn, 2011; Wynn et al., 2013). Therefore, it is not surprising that macrophage dysfunction may contribute to numerous pathologies and make this cell a prime target for therapeutic intervention. However, because of the central role of the macrophage in host defence, direct inhibition of macrophage functions is of limited value. A better therapeutic strategy would be to shape the macrophage actions, thus removing its aberrant effects while maintaining its desired functions. We suggest that the balance between macrophage pro-, anti-inflammatory and homeostatic responses is partly defined by ion channel actions. Therefore, could ion channel targeting drugs be used to fine-tune macrophage response in certain diseases?

Outlined above, a number of ion channels have been demonstrated to regulate macrophage biology, and a number of drugs which target these channels, predominantly in non-immune tissues/cells, have entered clinical trials or been approved for various diseases, e.g. epilepsy, cardiac dysrhythmia and anxiety (Imbrici et al., 2013; Priest and McDermott, 2015; Cojocaru et al., 2021) (Table 4). Illustrated in Table 4, drugs which target ion channels can be designed to block or open ion channels, as well as act as positive allosteric modulators. This together with various channel subunit compositions (both core and auxiliary) give researchers a large chemical space to explore when developing drugs targeting ion channels in macrophages and minimizing off target/tissue effects. Table 5 introduces the reader to several examples of diseases which may be amenable to targeting macrophage ion channels for their treatment.

**TABLE 5:** Therapeutic opportunities for macrophage ion channel-targeted drugs.

Channel	Modulation	Disease
Potassium (K^+^) ion channels	Inhibition	Atherosclerosis (Toyama et al., 2008) (Xu et al., 2017)
	(K_Ca_3.1 inhibitors such as TRAM-34 and NS6180, K_v_1.3 blockers such as ShK-170)	Inflammatory bowel disease (Strøbæk et al., 2013)
		Kawasaki Disease (Zheng et al., 2021)
		Glioma (Grimaldi et al., 2016)
		Brain injury (Mauler et al., 2004) (Peng et al., 2014)
		Ischemic stroke (Chen et al., 2011)
		Dementia (Chung et al., 2002) (Tsai et al., 2013)
		Rheumatoid arthritis (Vaiciuleviciute et al., 2021)
		Inflammatory bowel disease (Strøbæk et al., 2013)
Sodium (Na^+^) ion channels	Inhibition	Multiple sclerosis (Black et al., 2013)
		Encephalomyelitis (Craner et al., 2005)
		Atherosclerosis (Sun et al., 2019)
Transient receptor potential ion channels	Inhibition	Cardiogenic pulmonary edema (Kuebler et al., 2020) (Goyal et al., 2019)
	(TRPV4 antagonist GSK2798745 in clinical trials, TRPM2 blockers)	Chronic tuberculosis (Naik et al., 2020), Atherosclerosis (Zong et al., 2022)
	Activation	Osteoarthritis (Atobe, 2019; Atobe et al., 2019), Atherosclerosis (Xu et al., 2016) (Wang et al., 2020)
	(TRPA1 agonists such as cinnamaldehyde, TRPV4 and TRPML3 agonists)	Colitis (Kun et al., 2014), Cystic fibrosis (Riazanski et al., 2015), Chronic obstructive pulmonary disease? (Spix et al., 2022)
P2X receptor	Inhibition	Neuropathic pain (Ulmann et al., 2010) (Beggs et al., 2012) (Masuda et al., 2014)
Gamma-Aminobutyric Acid type A receptor	Inhibition	Pressure overload-induced heart failure? (Bu et al., 2021)
Proton ion channel	Inhibition	Ischemic stroke (Wu et al., 2012)
Piezo 1	Modulatory	Atherosclerosis (Umar Shinge et al., 2022)

## Conclusion

In this review we have highlighted the current state of the literature regarding the expression and roles of ion channels in macrophages (Table 1; Figure 1). We believe that targeting ion channels in macrophages represents a viable therapeutic opportunity for the treatment of a number of diseases (Table 5). Indeed, rather than inhibiting inflammatory responses, we believe targeting ion channels may be able to shape the inflammatory response and thus provide better clinical utility.
